# Defining optimal electrospun membranes to enhance biological activities of human endometrial MSCs

**DOI:** 10.3389/fbioe.2025.1551791

**Published:** 2025-02-26

**Authors:** Jiangru An, Tianyi Ma, Qiuhua Wang, Jinyi Zhang, J. Paul Santerre, Wenshuang Wang, Peng Ma, Xiaoqing Zhang

**Affiliations:** ^1^ International Joint Laboratory of Biomaterials and Tissue Regeneration, School of Basic Medicine, Binzhou Medical University, Yantai, Shandong, China; ^2^ Li Ka Shing Faculty of Medicine, The University of Hong Kong, Pokfulam, Hong Kong SAR, China; ^3^ Institute of Biomedical Engineering, University of Toronto, Toronto, ON, Canada; ^4^ Department of Gynecology, Yuhuangding Hospital, Yantai, Shandong, China

**Keywords:** human endometrial mesenchymal stem cells, biomaterial electrospun membranes, Poly-caprolactone, silk fibroin, hyaluronic acid

## Abstract

**Introduction:**

Human endometrial mesenchymal stem cells (H-EMSCs) can inhibit endometrial fibrosis and repair damaged endometrium. However, direct cell injection into dam-aged endometrium shows limited cell survival. Cell seeding onto biomaterial-based electrospun membranes could improve H-EMSCs’ survival and prolong their stay at the damaged endometrium. Polycaprolactone (PCL), silk fibroin (SF) and hyaluronic acid (HA) are synthetic or natural biomaterials used by the biomedicine field, however, their effects on the biological activities of H-EMSCs remain unclear.

**Methods:**

In this study, CD90^+^CD73^+^CD45^−^ H-EMSCs were extracted from human endometrium and H-EMSCs showed enhanced adhesion, proliferation on PCL-HA vs. PCL, PCL-SF, establishing the potential of the composite to address cell survival issues.

**Results:**

H-EMSCs cultured on PCL-HA showed decreased IL-6 gene expression and increased IL-10, VEGFA, TGF-β gene expression vs. PCL-SF, establishing the potential to create a favorable micro-environment for generating vascularized endometrial tissues. PCL, PCL-SF, PCL-HA all supported CD90 and Meflin expression of the seeded H-EMSCs, establishing PCL as a platform to form enhanced biomaterial composites for endometrial repair in the future.

**Discussion:**

This study provided significant evidence sup-porting the potential of appropriately tailored composites of PCL and HA to moder-ate inflammation and wound-healing, which can be applied for endometrial tissue repair and regeneration.

## 1 Introduction

Intrauterine adhesion (IUA) refers to the damage of the basal layer within the endometrium, due to miscarriage, infection, or medical injury, which can lead to partial or complete obstruction of the uterine cavity (Emingr et al., 2023). The main clinical symptoms of IUA include decreased menstrual volume, amenorrhea, infertility, repeated abortion, and periodic lower abdominal pain (Kou et al., 2020). At present, the gold-standard treatment for IUA is hysteroscopic adhesiolysis, for which intrauterine devices and intrauterine balloons can be inserted to dilate the narrow uterine cavity and improve the long-term prognosis of patients (Vitale et al., 2022). However, the effectiveness of these strategies is low, as they essentially rely on the natural self-repair ability of the endometrium, which can take a long time, rather than directly repairing the damaged endometrium (Liu et al., 2023). Studies have shown that human endometrial mesenchymal stem cells (H-EMSCs) have the ability of self-renewal and tissue regeneration, which could directly repair the damaged endometrium, reduce the extent of the endometrial fibrotic area, and increase the endometrial layer thickness (Zuo et al., 2018). However, the effect of direct injection of stem cells into the damaged endometrium is limited, as the survival rate of the transplanted stem cells into the damaged tissue is low and the cell retention time is short (Cen et al., 2022). It is hypothesized that if the cells could first establish stability, via forming a niche micro-environment on an extracellular matrix (ECM)-like constructs, such as a biodegradable biomaterial-based scaffold, then such a construct would provide an effective carrier for H-EMSCs, to increase the survival rate of the transplanted H-EMSCs and prolong the contact time between H-EMSCs and the damaged endometrium to achieve improved endometrial tissue repair (Cen et al., 2022).

Electrospinning has become one of the mainstream techniques for the preparation of nanofibrous materials and enabling the use of stem cells or tissue cells to generate tissue patches for tissue repair and wound healing (Hong et al., 2019). Electrospinning has advantages including ease of operation, low cost, and compatibility with biomaterials of different chemical and physical characteristics (Maurmann et al., 2023). In addition, the electrospun biomaterial membranes can be generated to resemble the structure of natural extracellular matrices and could be fabricated to have ultra-fine continuous fibers, high surface-volume ratio, high porosity and variable pore size distribution (Maurmann et al., 2023). The scaffold chemistry and structure could affect cell attachment, migration, proliferation and differentiation (Manoukian et al., 2017).

Endometrial repair is a complex and delicate physiological process involving the interaction of multiple cell types, growth factors, and extracellular matrix (ECM) components (Ma et al., 2021). In recent years, the development of biomaterials has provided new strategies for endometrial tissue repair, among which polycaprolactone (PCL), silk fibroin (SF) and hyaluronic acid (HA), as important synthetic/natural polymer biomaterials, have shown significant potential for endometrial tissue repair applications (Ayran et al., 2022; Lin et al., 2022; Zheng et al., 2022). PCL is an organic polymer approved by the Food and Drug Administration (FDA) of the United States and it has strong mechanical properties, good biocompatibility and is biodegradable over defined periods (Ayran et al., 2022). However, its hydrophobicity can limit cell adhesion and proliferation (Homaeigohar and Boccaccini, 2022). SF, which is a naturally-occurring high molecular weight protein extracted from silk, is mainly composed of glycine, alanine and serine (Debari et al., 2021). SF has been shown to be non-toxic, non-irritating, and it also has excellent flexibility, tensile resistance, moisture permeability, biodegradability, as well as having versatility in its structural organization (Sun et al., 2021). Because of its excellent biocompatibility, SF has been widely used in tissue engineering and wound healing applications (Kundu et al., 2013). In a previous study, researchers constructed a SF-small intestinal submucosa (SF-SIS) scaffold that was seeded with human umbilical cord mesenchymal stem cells (HUMSCs) and evaluated its ability to repair damaged endometrium in a mouse IUA model (Zheng et al., 2022). It was found that HUMSCs-SF-SIS scaffold increased the gland numbers and decreased inflammation and the damaged endometrial tissue area in this IUA model (Zheng et al., 2022). Additionally, another study incorporated Stromal Cell-Derived Factor-1α (SDF-1α) into SF-Cellulose membranes to repair endometrial damage in a rat IUA model (Cai et al., 2019). The results demonstrated that SDF-1α-loaded SF-Cellulose membranes significantly promoted the wound-healing, regeneration and angiogenesis of the damaged rat endometrium and improved the pregnancy outcomes (Cai et al., 2019). Another popular biomaterial is HA. HA is a natural linear anionic polysaccharide, derived from native ECM (Kotla et al., 2023), and has demonstrated excellent hydrophilicity, biocompatibility, biodegradability and non-immunoreactivity (Vasvani et al., 2020). HA is an important regulator of cell adhesion, proliferation and differentiation (Rashki Ghaleno et al., 2024). Recently, HA has become a popular biomaterial in endometrial repair. For instance, one study encapsulated human placenta-derived mesenchymal stem cells into HA hydrogels (Lin et al., 2022). It was found that the cell-loaded HA hydrogel can significantly increase angiogenesis, endometrial tissue cell proliferation, endometrium thickness, reduce the fibrotic tissue area and enhance the embryo implantation rate in a mouse endometrial damage model (Lin et al., 2022). Similarly, reduced endometrial fibrosis and inflammation, increased endometrial layer thickness as well as restored reproduction capability were demonstrated when endometrial stromal cells were mixed into HA-fibrin hydrogel and got injected into a mouse uterine infertility model (Kim et al., 2019). To explore whether this strategy can be applied to humans, a recent study established a non-human primate IUA model (using rhesus monkeys that have similar uterus structures to humans) to investigate the repairing effects of HA hydrogel combined with human UC-MSCs on endometrial injury and adhesion (Wang L. et al., 2020). The results showed decreased adhesion, increased endometrium thickness and gland number with the treatment, suggesting the UC-MSCs-HA hydrogel can promote endometrial tissue repair and regeneration (Wang L. et al., 2020). However, HA is rarely able to be used alone in tissue engineering due to its modest mechanical properties (Castro et al., 2021; Chen et al., 2023). SF is also often used in combination with lower cost synthetics which can tailor both mechanical and functional properties to enhance SF performance.

Therefore, PCL, which is a synthetic biomaterial that is often used to enhance mechanical properties and accommodate different degradation rates of composite materials (Ayran et al., 2022), can be combined with natural biomaterials such as SF and HA to generate hybrid biomaterials to potentially yield improved tissue repair and regeneration. Although seeding H-EMSCs onto PCL-based electrospun biomaterials to generate endometrial patches for treating IUA has potential merits, the influence of such electrospun membranes (i.e., PCL, PCL-SF, PCL-HA) on the adhesion, proliferation, and more importantly the pro-inflammatory, anti-inflammatory/wound-healing gene expression profile of H-EMSCs, have not been systematically investigated.

Therefore, this study aimed to isolate H-EMSCs from human endometrial tissue and evaluate the effects of a series of PCL-based electrospun membranes (PCL, PCL-SF and PCL-HA) on the adhesion, proliferation, and inflammatory and wound-healing genes’ expression profile of H-EMSCs. This study would provide significant insights into the influence of synthetic/natural biomaterial composites on H-EMSCs, the ultimate design of a biocompatible electrospun membrane for H-EMSCs seeding, as well as verifying the potential of an endometrial patch, fabricated by seeding H-EMSCs on a biocompatible electrospun membrane, in order to yield a strategy towards the repair of endometrial tissues post-injury and ultimately treating IUA.

## 2 Materials and methods

All materials were purchased from Sigma Aldrich unless stated otherwise.

### 2.1 Isolation and culture of H-EMSCs

Human endometrial tissue was obtained from Yuhuangding Hospital of Yantai (the protocol was approved by the ethical review committee of Binzhou Medical University, with an ethics approval number: 2023–043). A 50 mL centrifuge tube was prepared in advance, 15 mL PBS containing 1% penicillin/streptomycin was added to the tube and the human endometrial tissue samples were collected in the tube and transported on ice. The endometrial tissue was rinsed 3 times with PBS to remove blood. Tissues were cut into 1 mm³ pieces and digested with collagenase type I (1 mg/mL) for 60 min at 37°C within a constant temperature oscillator (80 r/min). DMEM/F12 complete medium (containing 10% FBS and 1% penicillin/streptomycin) was used to terminate the digestion. The cells were centrifuged at 1,000 r/min for 5 min and the cell pellet was re-suspended with DMEM/F12 complete medium and seeded into T75 TCPS culture flasks to obtain passage 1 H-EMSCs. Culture medium was changed 24 h immediately after seeding and then changed every 2–3 days in culture. When the H-EMSCs reached 80%–90% confluency, they were passaged using a ratio of 1:2. P4 H-EMSCs were used for all the experiments in this study, according to previous studies (Rahimipour et al., 2021; Grinchuk et al., 2023). The morphology of the H-EMSCs was observed by inverted microscope and representative pictures were taken.

### 2.2 Fabrication of PCL-based electrospun membranes

PCL, PCL:HA (80:20), PCL:SF (80:20) were added to 10 mL hexafluoro-isopropanol and the solutions were magnetically stirred for 12 h to obtain transparent spinning solutions. The solutions were poured into a syringe and the electrospinner (Ne300, Inovenso Inc.) was set to have a flow rate of 1.5 mL/h, a spinning voltage of 12.6 kV and a spinning distance of 20 cm. After electrospinning, the electrospun membranes were dried in a vacuum oven for 48 h to remove residual solvent and then stored in a desiccator in dark until use.

### 2.3 Scanning electron microscopy (SEM)

The H-EMSCs seeded electrospun membranes were rinsed with PBS and fixed with 2.5% glutaraldehyde (Electron Microscopy Sciences) overnight at 4°C. The samples were dehydrated with 30%, 50%, 70%, 80%, 90%, 100% ethanol gradient (15 min each) and sprayed with gold. Representative pictures were taken using a scanning electron microscope (Carl Zeiss AG). The representative SEM images were analyzed using ImageJ software (Fiji, version J1.46r., NIH) to assess the electrospun fiber diameters.

### 2.4 Degradation assay

6 mm-diameter disc-shaped PCL, PCL-SF and PCL-HA electrospun membranes were immersed into a 15 mL falcon tube containing 10 mL of H-EMSCs culture supernatant. The tubes were incubated at 37°C for 0, 3, 7, 14 days with the cell culture supernatant changed twice a week for determining the biomaterials’ degradation rates. Degradation % = (W_0_-W_d_)/W_0_*100%, where W_0_ is the initial weight of the electrospun membrane and W_d_ is the dry weight of the electrospun membrane after the degradation test.

### 2.5 Porosity analysis of electrospun membranes

The porosity of the PCL, PCL-SF and PCL-HA electrospun membranes was determined using the following equations: porosity = (1-apprent density of electrospun membranes/bulk density of electrospun membranes)*100%, apparent density = mass of electrospun membranes/membrane thickness*membrane area. The membrane thickness was measured *via* cutting of membrane viewing of cross section under microscope.

### 2.6 Cell doubling assay

The H-EMSCs were seeded in 24-well plates at a density of 20,000 cells/well. H-EMSC numbers were counted and recorded after 48, 72 and 96 h in culture. Cell doubling time was determined with the following formula: Doubling time = (T-T_0_)*log2/(log N-log N_0_). T_0_ and T represent the start time and end time of cell culture, while N_0_ and N are the numbers of cells obtained at time T_0_ and T.

### 2.7 Flow cytometry

A 50 μL H-EMSC suspension was added into the flow tube, mixed with CD90-PE, CD73-PE-cy7, CD45-APC-cy7 antibodies, and incubated at 4°C for 40 min in the dark. After the incubation, the cells were washed with PBS, and then examined by flow cytometer (BD LSRFortessa™ X-20). Flowjo software was used for flow cytometry data analysis. Specifically, the flow files were open with Flowjo, the x-axes were changed to the corresponding channels and the y-axes were set to be histograms. The linear gate was set based on the blank group, and the box-selected part was copied to the experimental group to obtain the experimental group results, and finally the results were exported from Flowjo.

### 2.8 H-EMSC tri-lineage differentiation

Adipogenic differentiation: H-EMSCs were seeded in 96-well plates and after the H-EMSCs reached 80% confluency, lipogenic induction medium (10% FBS, 1% glutamine, 1% penicillin/streptomycin, 0.2% insulin, 0.2% IBMX, 0.1% rosiglitazone, 0.1% dexamethasone) was added and changed every 2 days. After 21 days of induction, the cells were stained with oil red O dye solution and observed with an inverted microscope.

Osteogenic differentiation: H-EMSCs were seeded in 96-well plates and after the H-EMSCs reached 80% confluency, osteogenic induction medium (10% FBS, 1% glutamine, 1% penicillin/streptomycin, 0.2% ascorbic acid, 0.01% dexamethasone, 1% sodium β-glycerophosphatase) was added and changed every 2 days. After 21 days of induction, the cells were stained with alizarin red dye solution and observed with an inverted microscope.

Chondrogenic differentiation: H-EMSCs were seeded in 96-well plates, and after the H-EMSCs reached 80% confluency, chondrogenic induction medium (10% FBS, 1% penicillin/streptomycin, 0.1% proline, 0.3% ascorbic acid, 1% TGF-β1, 1% sodium pyruvate, 1% ITS additive) was added and changed every 2 days. After 21 days of induction, the cells were stained with Alcian blue solution and observed with an inverted microscope.

### 2.9 Cell colony-forming assay

H-EMSCs were seeded in 6-well plates at a density of 40 cells/well. DMEM/F12 complete culture medium (16.5% FBS, 2 mM L-glutamine, 1% penicillin/streptomycin) was added and the cells were cultured for 14 days. At the end of cell culture, the cells were fixed with 4% paraformaldehyde for 20 min, stained with 3% crystal violet for 10 min and the representative colony pictures were taken with an inverted microscope.

### 2.10 H-EMSC seeding on electrospun membranes

6 mm-diameter disc-shaped PCL, PCL-SF and PCL-HA electrospun membranes were placed into a 48-well plate. 70% ethanol was added to each well to sterilize the electrospun membranes for overnight, and then PBS was added to rinse the membranes three times until all residual ethanol was removed. Cells were seeded on the PCL, PCL-SF and PCL-HA electrospun membranes, as well as TCPS (relative control condition) at a density of 50,000 cells/well, and cultured for 1, 3, and 7 days to study the effects of different biomaterial-based electrospun membranes on the adhesion, proliferation and pro-inflammatory and anti-inflammatory gene expression activities of H-EMSCs.

### 2.11 DNA quantification assay and WST-1 analysis

DNA quantification assay: After 1, 3, and 7 days of culture, 50 μL 0.05% Triton/EDTA cell lysis buffer (Solarbio) was added to each well to lyse the cells on ice for 1 h. The sample was then vortexed for 30 s and incubated at 65°C for 30 min (two times in total) to complete the lysis and then centrifuged at 4°C for 15 min at 15,000 rpm. After obtaining the sample solution, the DNA content for all samples were quantified by binding with Hoechst 33,258 dye (1 mg/mL, Solarbio). The test samples and the calf thymus DNA standards of known concentrations were read using a plate reader (SpectraMax M2) with excitation at 360 nm and emission at 460 nm.

WST-1 analysis: After 1, 3, and 7 days of culture, 1:10 WST-1 reagent (Beyotime): DMEM/F12 complete medium was added into the H-EMSC culture and incubated at 37°C for 1 h. The absorbance was measured at 450 nm (SpectraMax M2).

### 2.12 Phalloidin staining

H-EMSCs cultured on different electrospun membranes were fixed with 4% paraformaldehyde for 10 min at room temperature. Then, cells were washed three times with PBS for 10 min each time. After that, H-EMSCs were permeabilized with 0.5% Triton X-100 solution for 5 min and then incubated with 200 μL TRITC labeled phalloidin working solution (2 μL phalloidin storage solution, 198 μL PBS) at room temperature in the dark for 30 min. After the incubation, cells were washed three times with PBS for 5 min each time. Nuclei were counterstained using 200 μL DAPI solution for about 30 s. The cells seeded on the different electrospun membranes were observed under a fluorescence microscope (EVOS Floid, Thermo Fisher Scientific Inc.) and representative pictures were taken. In addition, confocal imaging (Zeiss LSM710 Two-Photon/Confocal, AOMF) was also performed for the H-EMSCs cultured electrospun membranes.

### 2.13 qRT-PCR

mRNA was extracted from the cells using the Trizol (Vazyme) method. mRNA quality and quantity were checked with NanoDrop™ 1,000 Spectrophotometer (Thermo Scientific), and the samples were stored in −80°C freezer. cDNA was synthesized using the reverse transcription kit (Vazyme), according to the manufacturer’s instructions. The obtained cDNA samples were diluted 10 times with nuclease free water and used for qRT-PCR. Reaction mixtures containing 10 μL of two x ChamQ SYBR qPCR Master Mix, 0.4 μL forward primer (10 μM), 0.4 μL reverse primer (10 μM), 0.7 μL cDNA sample, 8.5 μL ddH_2_O were prepared. The qPCR reaction was performed with Roche LightCycler™ Real-Time PCR Detection System, using the following protocol: Pre-incubation: 95°C for 10 min, Amplification (40 cycles): 95°C for 10 s, 60°C for 30 s, Melting curve: 95°C for 15 s, 60°C for 1 min, 95°C for 15 s. The data was analyzed using the comparative 2^−ΔΔCt^ method. The forward and reverse primer sequences of the genes can be found in the following Table 1.

**TABLE 1:** The forward and reverse primer sequences of the genes (CD90, Meflin, VEGFA, VEGFB, TGF-β, IL-10, IL-6).

CD90	Forward (5′–3′): CGCCTTCACTAGCAAGGACGAG Reverse (5′–3′): CTGATGCCCTCACACTTGACCA
Meflin	Forward (5′–3′): CTGGACCTCAGCCACAATCTCA Reverse (5′–3′): CAGCTCGTTGCTGTCCATCTTG
VEGFA	Forward (5′–3′): GAGCCTTGCCTTGCTGCTCTA Reverse (5′–3′): CACCAGGGTCTCGATTGGATG
VEGFB	Forward (5′–3′): GCTTAGAGCTCAACCCAGACACC Reverse (5′–3′): CAAGTCACCCTGCTGAGTCTGAA
TGF-β	Forward (5′–3′): GCCGACTACTACGCCAAGGA Reverse (5′–3′): ATGCTGTGTGTACTCTGCTTGAAC
IL-10	Forward (5′–3′): CTTGCTGGAGGACTTTAAGGGTTA Reverse (5′–3′): CTTGATGTCTGGGTCTTGGTTCT
IL-6	Forward (5′–3′): CCAGTTCCTGCAGAAAAAGGCA Reverse (5′–3′): AGCTGCGCAGAATGAGATGAGT

### 2.14 Data analysis

Statistical analysis was performed using SPSS Statistics 22.0 software (SPSS Inc., Chicago, IL) by analysis of variance (ANOVA) using Tukey for pair-wise comparisons or an independent samples t-test where appropriate, with statistical significance reported for *p* < 0.05. Tests for homogeneity of variance (Leven’s test) and if the data were normally distributed were performed in SPSS to ensure the assumptions inherent to the statistical tests were valid. All experiments were repeated at least three times with at least three samples each time (N = 3, n = 3), unless stated otherwise. Data was represented as mean ± S.E.M.

## 3 Results

### 3.1 Characterization of the isolated H-EMSCs

**FIGURE 1:**
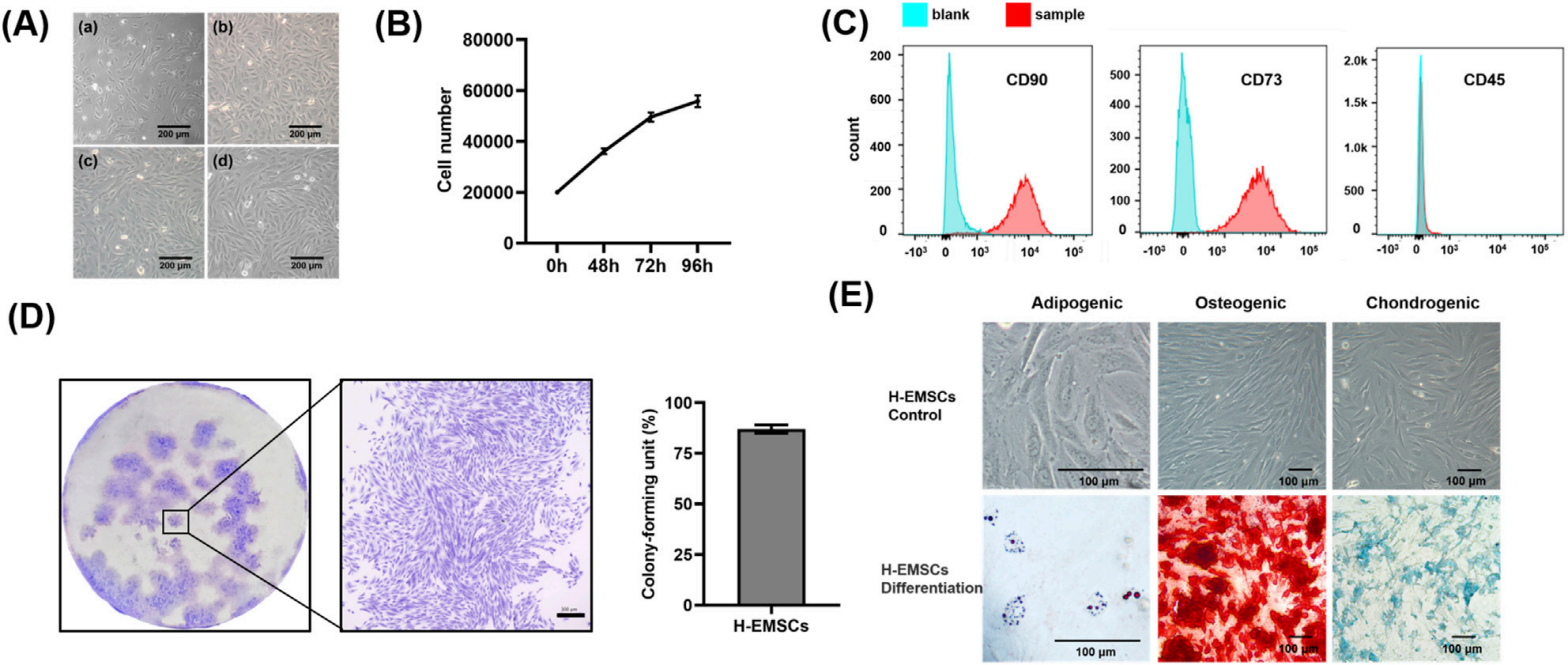
Characterization of isolated H-EMSCs. **(A)** Morphology of H-EMSCs of passage 1 (a), passage 2 (b), passage 3 (c) and passage 4 (d), Scale bar = 200 μm; **(B)** H-EMSC growth curve; **(C)** Flow cytometry of H-EMSCs (CD90, CD73 and CD45); **(D)** Colony formation of H-EMSCs (distribution of the H-EMSC colonies; a representative H-EMSC colony, scale bar = 300 μm; the colony-forming unit (%) of H-EMSCs); **(E)** Adipogenic, osteogenic and chondrogenic differentiation of H-EMSCs, the cells were stained using oil red О, Alizarin red and Alcian blue respectively. Scale bar = 100 µm. Statistical analysis was performed using SPSS Statistics 22.0 software (SPSS Inc., Chicago, IL) by analysis of variance (ANOVA) using Tukey for pair-wise comparisons. Tests for homogeneity of variance (Leven’s test) and if the data were normally distributed were performed in SPSS to ensure the assumptions inherent to the statistical tests were valid. N = 3, n = 3. Data = mean ± S.E.M.

It can be seen that H-EMSCs showed an elongated spindle-like structure in culture, and the fourth passage H-EMSCs showed evidence of spiral growth form (Figure 1A). In addition, it was found that the cell doubling time was 55.93 h ± 2.15 h (between the 0 h and 72 h time points data) and the number of H-EMSCs continued to increase within the time period of 0–96 h (Figure 1B). However, the cell growth slowed down from 72 h to 96 h (Figure 1B).

In terms of the immunophenotype analysis of H-EMSCs (Figure 1C), it was found that CD90 and CD73 were highly expressed (96.5% ± 0.38%, 99.7% ± 0.32%) by the H-EMSCs, while CD45 was minimally expressed (1.45% ± 0.09%). Moreover, H-EMSCs demonstrated a strong colony forming ability, with a colony-forming unit efficiency of 87% ± 2.08% (Figure 1D). Further, H-EMSCs showed successful differentiation along the adipogenic, osteogenic and chondrogenic pathways (Figure 1E), as evidenced by the formation of red round bright lipid droplets (positive Oil red О staining), mineralized nodules (positive alizarin red staining), as well as glycosaminoglycan secretion (positive alcian blue staining).

### 3.2 Characterization of different electrospun membranes and the proliferation and metabolic activity of H-EMSCs on them

**FIGURE 2:**
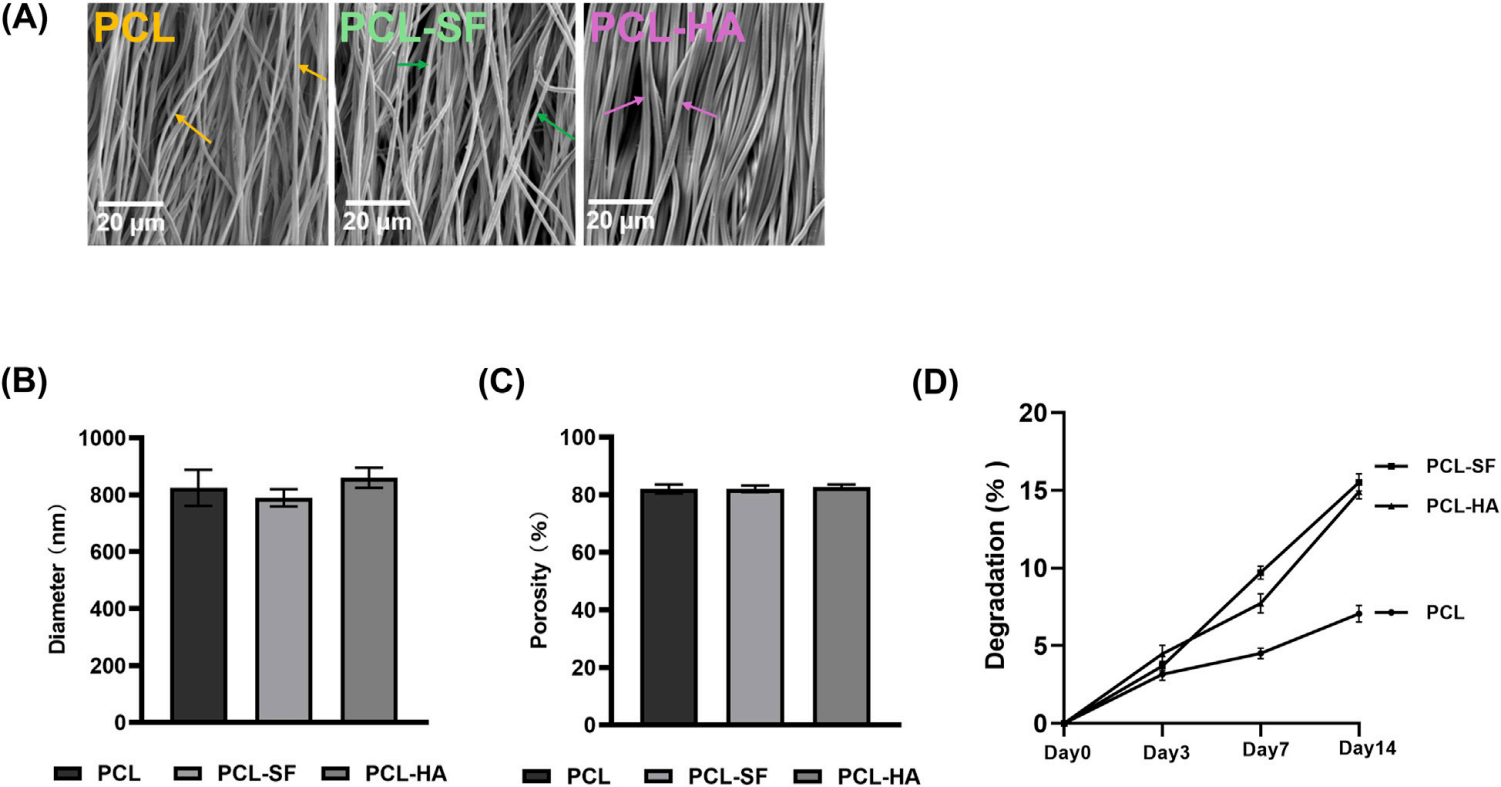
Characterization of PCL, PCL-SF, PCL-HA electrospun membranes. **(A)** Scanning electron microscopy of PCL, PCL-SF, PCL-HA electrospun membranes without H-EMSC seeding. Scale bar = 20 µm. **(B)** Fibre diameter quantification of PCL, PCL-SF and PCL-HA electrospun membranes. **(C)** Porosity (%) analysis of PCL, PCL-SF and PCL-HA electrospun fibres. **(D)** Degradation analysis of PCL, PCL-SF and PCL-HA electrospun fibres. Statistical analysis was performed using SPSS Statistics 22.0 software (SPSS Inc., Chicago, IL) by analysis of variance (ANOVA) using Tukey for pair-wise comparisons. Tests for homogeneity of variance (Leven’s test) and if the data were normally distributed were performed in SPSS to ensure the assumptions inherent to the statistical tests were valid. N = 3, n = 3. Data = mean ± S.E.M. Yellow arrows indicate representative fibers of PCL electrospun membrane, green arrows indicate representative fibers of PCL-SF electrospun membrane and pink arrows indicate representative fibers of PCL-HA electrospun membrane.

**FIGURE 3:**
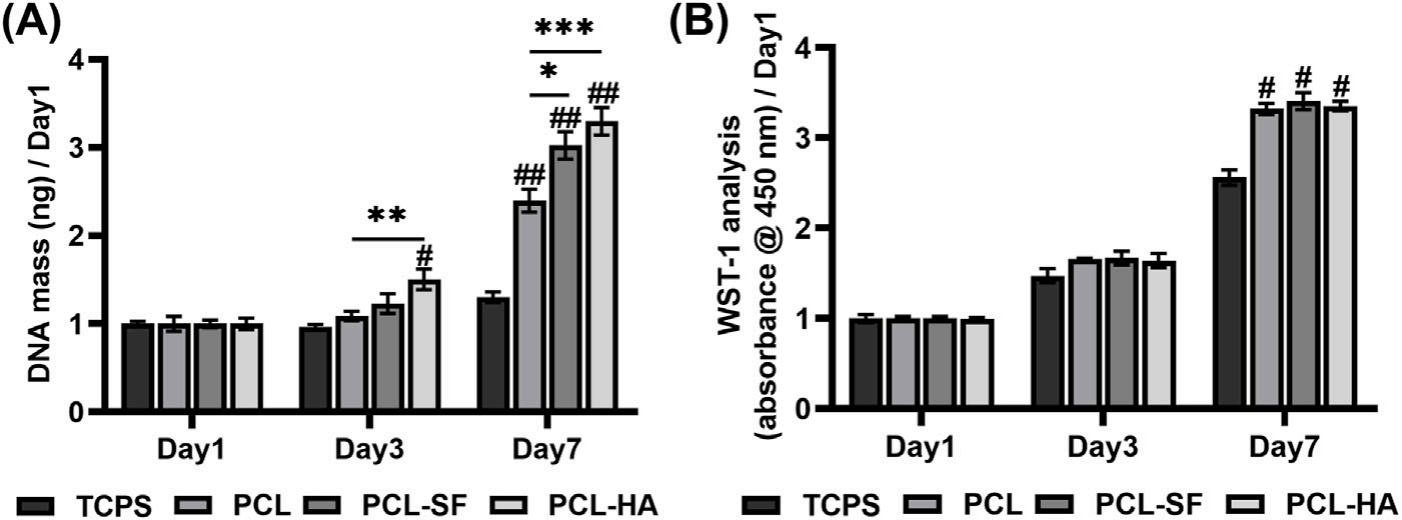
DNA quantification and WST-1 analysis of H-EMSCs seeded on TCPS and PCL, PCL-SF, PCL-HA electrospun membranes at day 1, day 3 and day 7 (normalized to day 1). **(A)** DNA quantification. *p < 0.05, **p < 0.01, ***p < 0.001. #*p* < 0.01, ##*p* < 0.001 vs TCPS. **(B)** WST-1 analysis. #*p* < 0.001 vs TCPS. Statistical analysis was performed using SPSS Statistics 22.0 software (SPSS Inc., Chicago, IL) by analysis of variance (ANOVA) using Tukey for pair-wise comparisons. Tests for homogeneity of variance (Leven’s test) and if the data were normally distributed were performed in SPSS to ensure the assumptions inherent to the statistical tests were valid. N = 3, n = 3. Data = mean ± S.E.M.

The scanning electron microscopy of PCL, PCL-SF, PCL-HA electrospun membranes without H-EMSC seeding can be seen in Figure 2A. The three different electrospun membranes showed similar fibre diameter and porosity (Figures 2B, C), however, PCL-SF and PCL-HA showed faster degradation rate vs PCL over the 14-day degradation study period (Figure 2D). SEM images of PCL, PCL-SF and PCL-HA after degradation (14 days) can be found in Supplemental Figure 1. The effects of different electrospun membranes (PCL, PCL-SF, PCL-HA) on the proliferation and metabolic activities of H-EMSCs were investigated using the DNA quantification and WST-1 assays (Figure 3). It can be seen that at day 3, the number of H-EMSCs was significantly higher when they were seeded on PCL-HA vs TCPS or PCL (Figure 3A). At day 7, there were higher numbers of H-EMSCs on PCL, PCL-SF, PCL-HA electrospun membranes vs TCPS, and H-EMSCs showed higher proliferation on PCL-SF and PCL-HA hybrid electrospun membranes vs PCL alone membrane (Figure 3A). Additionally, it can be seen that the PCL, PCL-SF, PCL-HA electrospun membranes supported higher H-EMSCs’ metabolic activities at day 7 vs TCPS (Figure 3B).

### 3.3 The adhesion and distribution patterns of H-EMSCs seeded on different electrospun membranes

**FIGURE 4:**
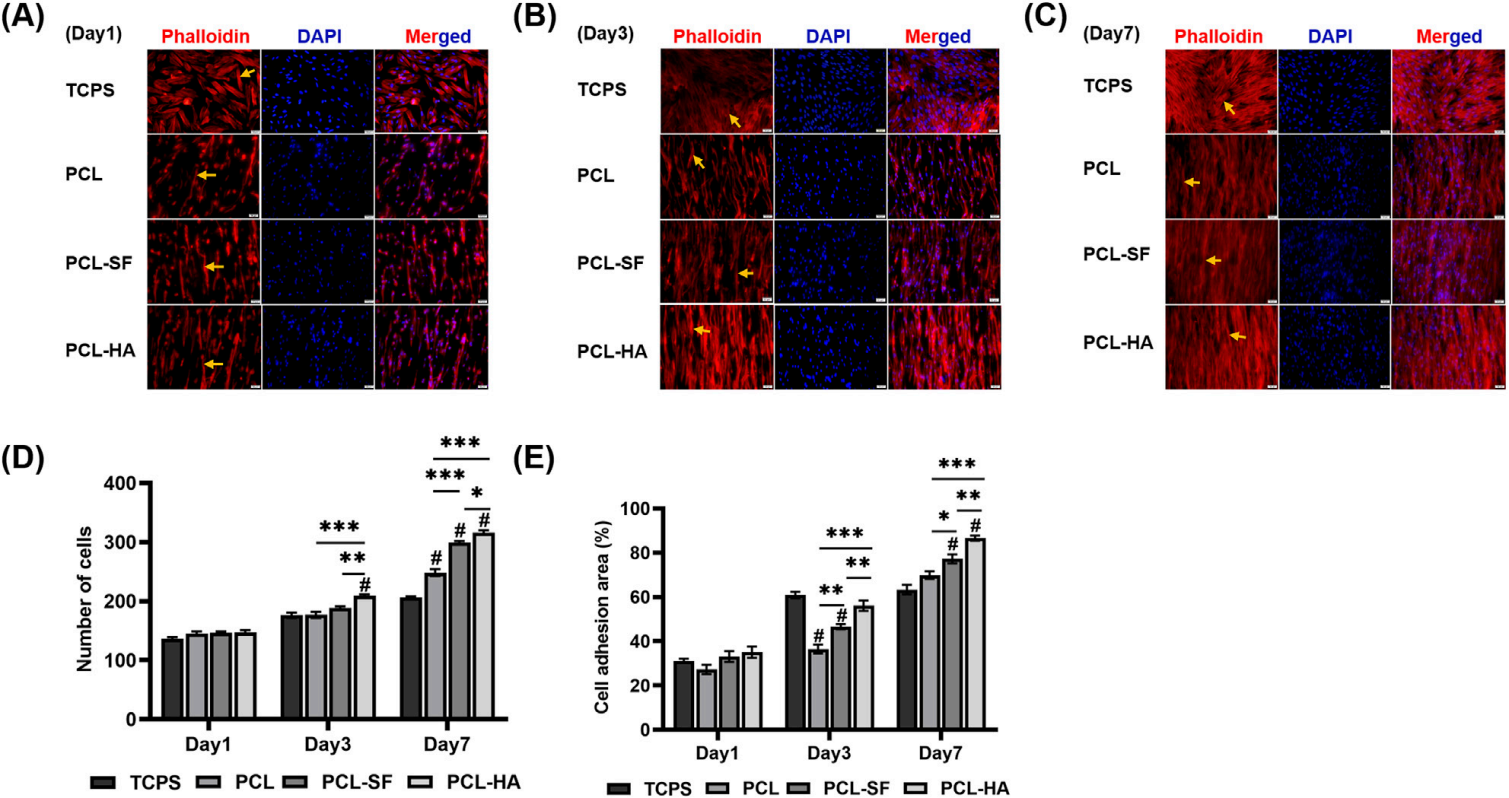
Phalloidin staining of H-EMSCs seeded on TCPS and PCL, PCL-SF, PCL-HA electrospun membranes at day 1, 3 and 7. Scale bar = 50 µm. **(A)** Phalloidin staining of H-EMSCs at day 1. **(B)** Phalloidin staining of H-EMSCs at day 3. **(C)** Phalloidin staining of H-EMSCs at day 7. **(D)** Cell number quantification. *p < 0.05, **p < 0.01, ***p < 0.001. #*p* < 0.001 vs TCPS. **(E)** % Cell adhesion area quantification. *p < 0.05, **p < 0.01, ***p < 0.001. #*p* < 0.001 vs. TCPS. Statistical analysis was performed using SPSS Statistics 22.0 software (SPSS Inc., Chicago, IL) by analysis of variance (ANOVA) using Tukey for pair-wise comparisons. Tests for homogeneity of variance (Leven’s test) and if the data were normally distributed were performed in SPSS to ensure the assumptions inherent to the statistical tests were valid. N = 3, n = 3. Data = mean ± S.E.M. Yellow arrows indicate representative H-EMSCs’ cytoskeletons on the different electrospun membranes **(A–C)**.

**FIGURE 5:**
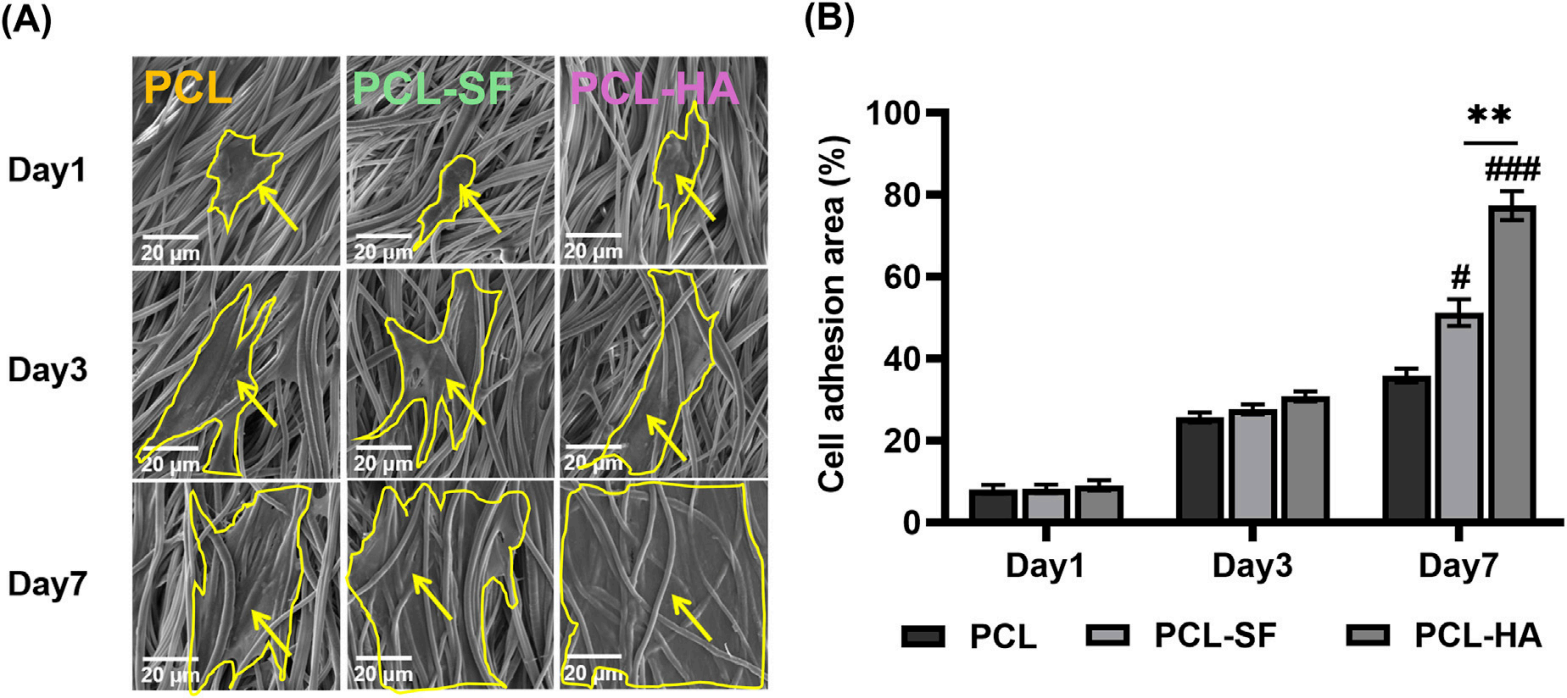
Scanning electron microscopy of H-EMSCs seeded on PCL, PCL-SF, PCL-HA electrospun membranes at day 1, day three and day 7. **(A)** Representative pictures showing the adhesion and infiltration of H-EMSCs on the different biomaterials. Scale bar = 20 μm, yellow arrows indicate H-EMSCs and the cells are circled with yellow lines. **(B)** Cell adhesion area (%) quantification. **p < 0.01. #*p* < 0.05, ###*p* < 0.001 vs PCL. Statistical analysis was performed using SPSS Statistics 22.0 software (SPSS Inc., Chicago, IL) by analysis of variance (ANOVA) using Tukey for pair-wise comparisons. Tests for homogeneity of variance (Leven’s test) and if the data were normally distributed were performed in SPSS to ensure the assumptions inherent to the statistical tests were valid. N = 4, n = 3. Data = mean ± S.E.M.

**FIGURE 6:**
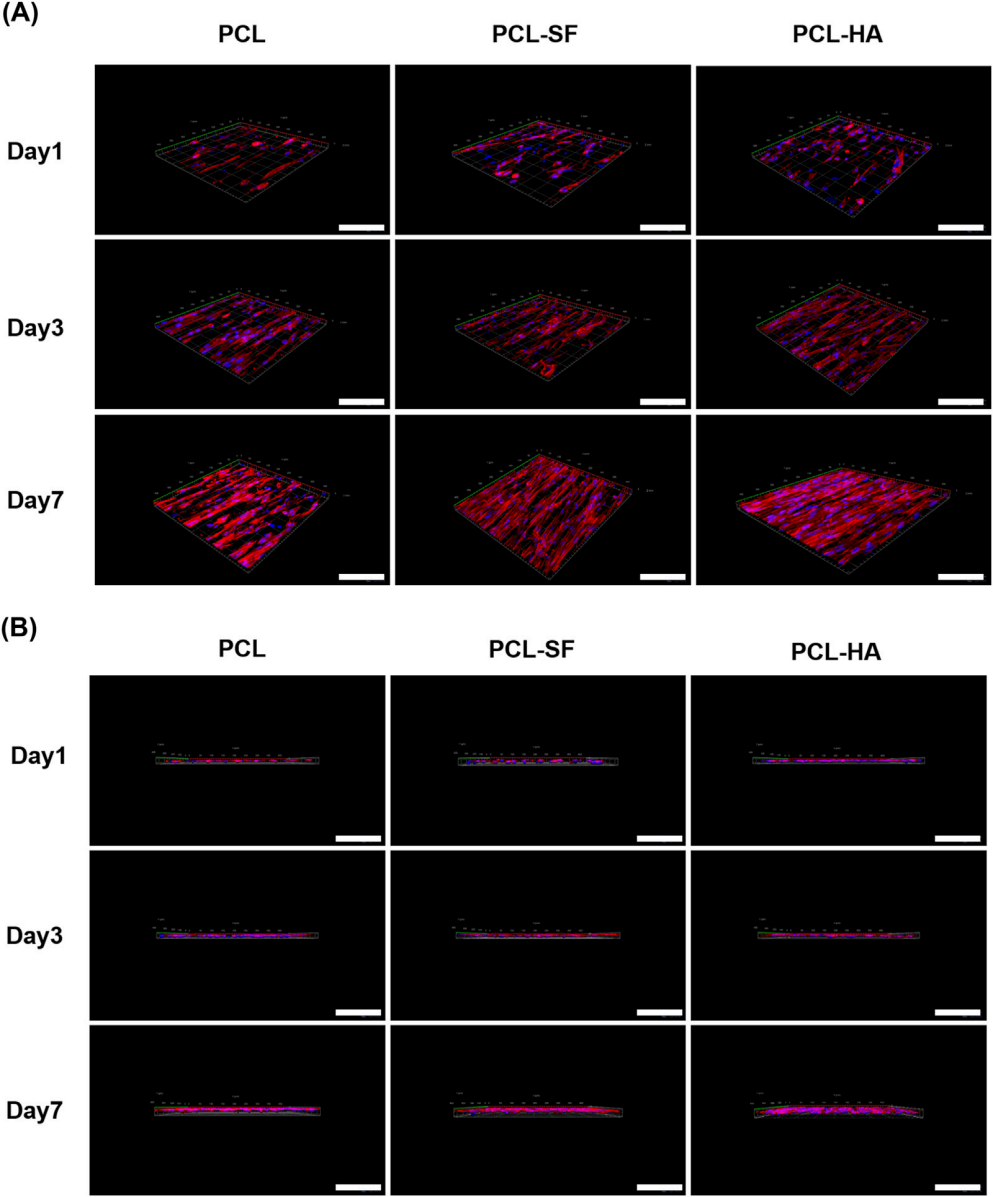
Confocal imaging of H-EMSCs seeded on PCL, PCL-SF, PCL-HA electrospun membranes. **(A)** Confocal imaging in the X-Y plane. **(B)** Confocal imaging in the Z-direction. N = 4, n = 3. Scale bar = 5 μm.

The effects of the different electrospun membranes (PCL, PCL-SF, PCL-HA) on the adhesion and distribution patterns of H-EMSCs were studied using Phalloidin staining and scanning electron microscopy (Figures 4, 5). The results showed that the hybrid PCL-SF and PCL-HA electrospun membranes supported greater adhesion and proliferation of H-EMSCs at day 7 vs TCPS and PCL (Figure 4), which matched the DNA quantification data. Those results indicated that the electrospun PCL-SF and PCL-HA membranes had good biocompatibility with H-EMSCs and promoted H-EMSC adhesion and proliferation, in comparison with the PCL alone membrane. In addition, PCL, PCL-SF, and PCL-HA electrospun membranes all supported H-EMSCs’ growth in the parallel direction along the electrospun fibers, while H-EMSCs on flat surface TCPS were distributed in random directions (Figure 4). In addition, comparing with the PCL-SF electrospun membrane, PCL-HA membranes supported greater H-EMSC adhesion and proliferation (Figure 4), indicating that the presence of HA could have provided hydrophilic and ionic functional groups to the material, to support more favorable adhesion and proliferation of H-EMSCs. Moreover, the scanning electron microscopy data also showed that PCL-HA supported enhanced H-EMSC adhesion vs PCL-SF at day 7, matching the Phalloidin staining data (Figure 5). Furthermore, confocal imaging of H-EMSCs seeded on PCL, PCL-SF, PCL-HA electrospun membranes was performed. It can be seen that the H-EMSCs aligned well with the direction of the electrospun fibers and the cells penetrated the entire depth of the electrospun membranes (Figure 6), confirming the uniform distribution of the cells within the electrospun biomaterial scaffolds.

### 3.4 The expression of mesenchymal stem cell genes, proinflammatory/wound-healing genes of H-EMSCs seeded on different electrospun membranes

**FIGURE 7:**
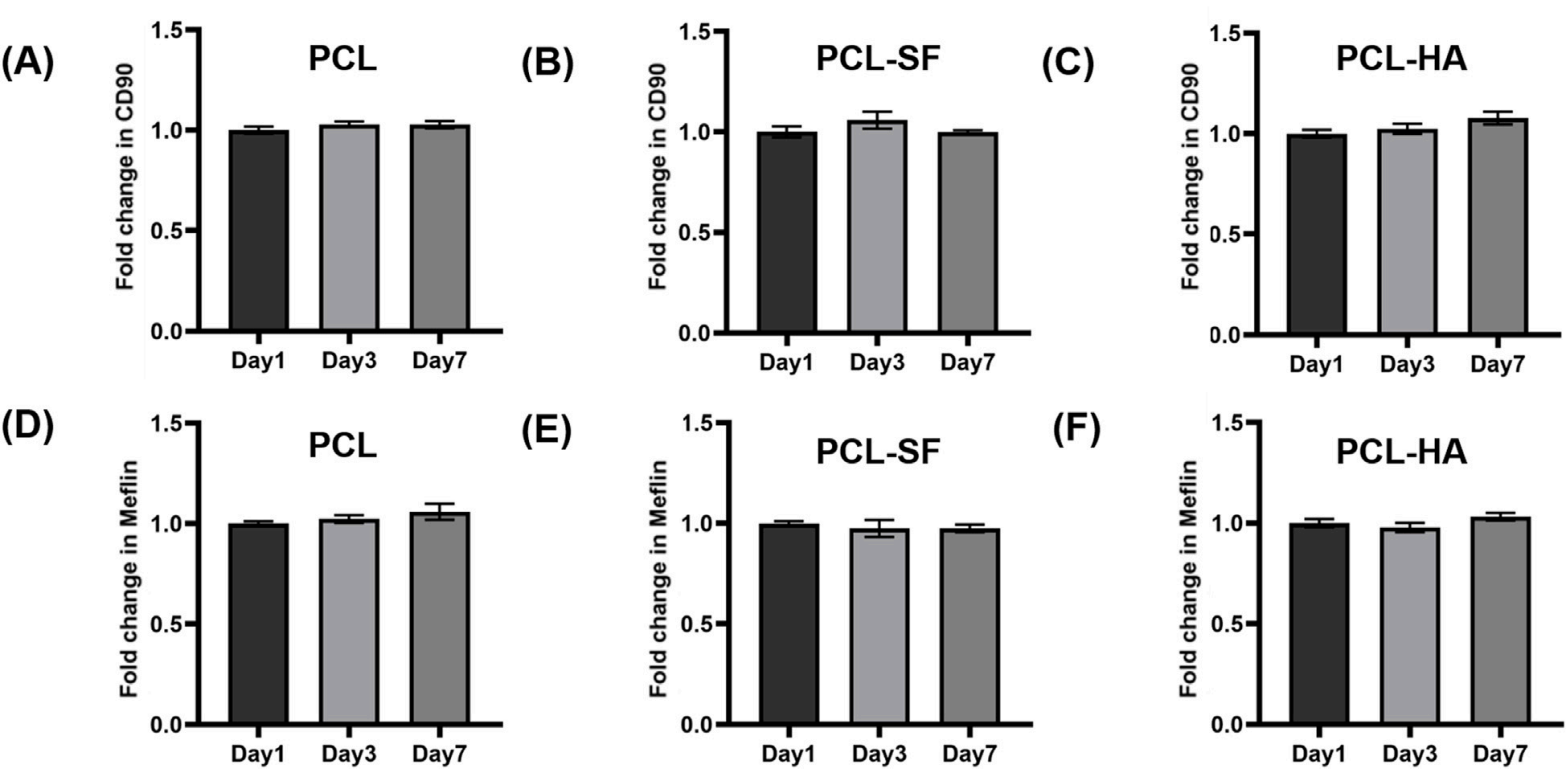
CD90 **(A–C)**, Meflin **(D–F)** gene expression of H-EMSCs seeded on PCL, PCL-SF, PCL-HA electrospun membranes (normalized to day 1). Statistical analysis was performed using SPSS Statistics 22.0 software (SPSS Inc., Chicago, IL) by analysis of variance (ANOVA) using Tukey for pair-wise comparisons. Tests for homogeneity of variance (Leven’s test) and if the data were normally distributed were performed in SPSS to ensure the assumptions inherent to the statistical tests were valid. N = 3, n = 3. Data = mean ± S.E.M.

The different electrospun membranes (PCL, PCL-SF, PCL-HA) were assessed to determine if they can influence the mesenchymal stem cell phenotype of H-EMSCs and their proinflammatory/wound-healing gene expression patterns. As can be seen in Figure 7, all three types of electrospun membranes (PCL, PCL-SF, PCL-HA) supported the mesenchymal stem cell genes CD90 and Meflin expression of H-EMSCs, suggesting that the electrospun membranes can maintain the mesenchymal stem cell phenotype of H-EMSCs during culture.

**FIGURE 8:**
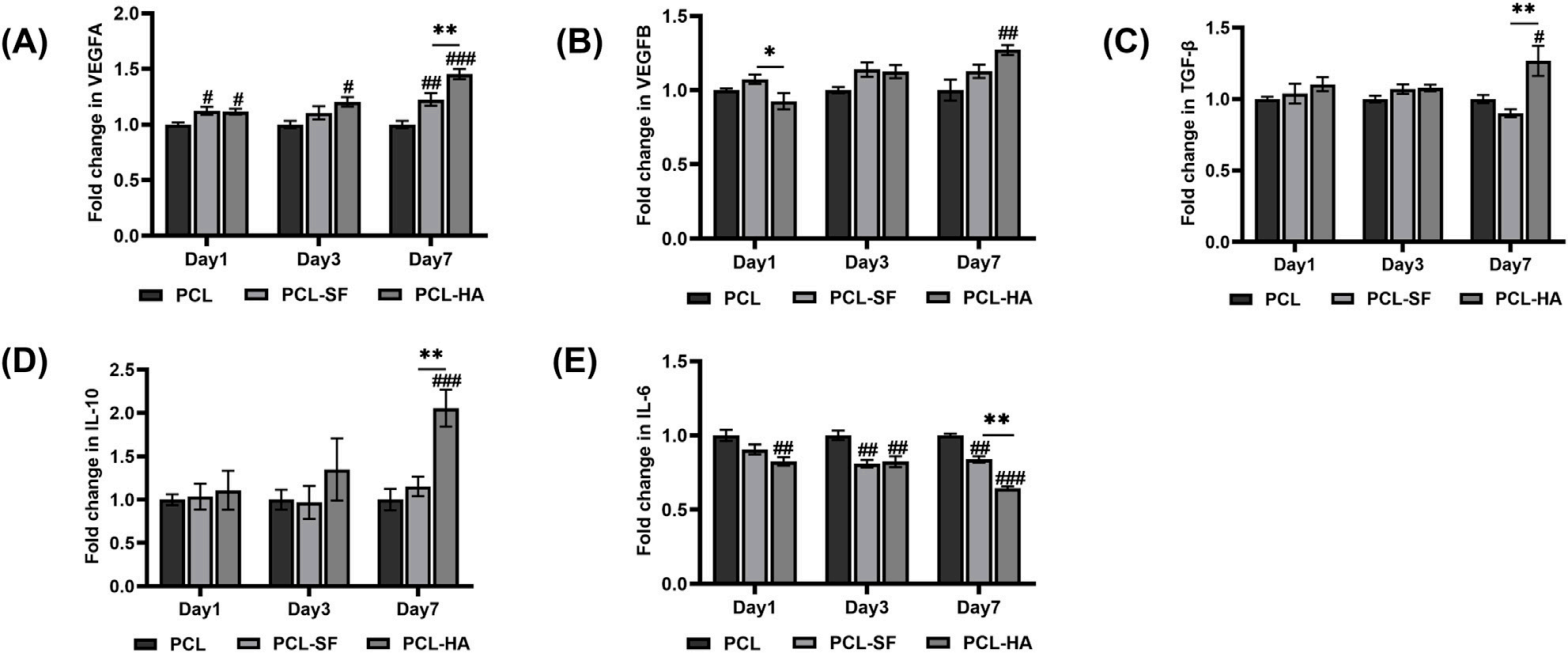
VEGFA **(A)**, VEGFB **(B)**, TGF-β **(C)**, IL-10 **(D)**, IL-6 **(E)** gene expression of H-EMSCs seeded on PCL, PCL-SF, PCL-HA electrospun membranes (normalized to day 1). *p < 0.05, **p < 0.01. #*p* < 0.05, ##*p* < 0.01, ###*p* < 0.001 vs PCL. Statistical analysis was performed using SPSS Statistics 22.0 software (SPSS Inc., Chicago, IL) by analysis of variance (ANOVA) using Tukey for pair-wise comparisons. Tests for homogeneity of variance (Leven’s test) and if the data were normally distributed were performed in SPSS to ensure the assumptions inherent to the statistical tests were valid. N = 3, n = 3. Data = mean ± S.E.M.

Although the different types of biomaterials (PCL, PCL-SF and PCL-HA) did not influence the mesenchymal stem cell gene CD90 and Meflin expression of the H-EMSCs cultured on them, they significantly affected the cells’ proinflammatory (IL-6), anti-inflammatory (IL-10) and wound-healing gene (VEGFA, VEGFB, TGF-β) expression patterns (Figure 8). It can be seen that PCL-HA significantly enhanced VEGFA expression vs PCL at day 3 and 7 time points (Figure 8A). In addition, the VEGFA expression of H-EMSCs was higher on PCL-HA vs PCL-SF at day 7 (Figure 8A). Similarly, PCL-HA also supported higher VEGFB and TGF-β expression of H-EMSCs, when compared with PCL at day 7 (Figures 8B, C). Moreover, anti-inflammatory gene IL-10 expression was also higher when the H-EMSCs were seeded on PCL-HA vs PCL or PCL-SF at day 7 (Figure 8D). Likewise, it was important to observe that PCL-SF and PCL-HA decreased IL-6 expression of H-EMSCs vs PCL at day 3 and 7 time points and comparing the H-EMSCs seeded on PCL-HA and PCL-SF, the former showed lower IL-6 expression than the later at day 7 (Figure 8E).

## 4 Discussion

The endometrium is the epithelial tissue on the surface of the uterine cavity, which forms during menstruation when a woman is not pregnant, and the endometrial cells can rapidly proliferate during pregnancy to provide the proper environment for the embryo (Wang Y. et al., 2020). The endometrium plays an important role in embryo implantation, specifically supporting pregnancy maintenance, and is thereby an essential element of human reproduction (Ng et al., 2020). Factors such as repeated curettage, cesarean section, myomectomy, or infection can cause the endometrium to fail to regenerate and form scars, leading to irregular menstruation, amenorrhea, abortion, infertility and other serious consequences (Ang et al., 2023). Conventional clinical methods have yielded limited efficacy, and they are ineffective in resolving the challenge of promoting endometrial tissue regeneration in patients with severe endometrial layer damage (Liu et al., 2023).

In recent years, mesenchymal stem cells have attracted worldwide attention due to their great potential in immune regulation and therapeutic functions in tissue regeneration (Arabpour et al., 2021). Mesenchymal stem cells can migrate to areas of tissue injury to modulate the immune cells in the tissue micro-environment, secrete anti-inflammatory cytokines, reconstruct or promote wound healing (Fujii and Miura, 2022). Studies have found that H-EMSCs-based therapies have achieved some promising results in the treatment of IUA, since H-EMSCs have demonstrated self-renewal ability, low immunogenicity and low tumorigenicity (Bozorgmehr et al., 2020). The experimental results of this study showed that the H-EMSCs can be successfully isolated from adult human endometrial tissue. The H-EMSCs showed classical elongated spindle-like mesenchymal stem cell morphology by passage 4, expressed mesenchymal stem cell markers (CD90 and CD73) at high levels, and had strong tri-lineage differentiation (adipogenic, osteogenic, and chondrogenic) potential. In addition, the H-EMSCs isolated in this study also revealed great colony formation capability.

However, the direct injection of stem cells into the damaged endometrium has demonstrated limited efficacy, and the survival rate of transplanted stem cells in the damaged tissue is very low (Benor et al., 2020). Adverse factors such as inflammation and oxidative stress within the local tissue environment can interfere with the survival ability of transplanted stem cells in injured tissue (Li et al., 2021). Studies have found that biomaterials can improve the viability of stem cells and prolong the contact time between stem cells and damaged tissues, enabling better repair potential (Zhang et al., 2013). Properly configured biomaterials for both physical structure and chemical composition can also mimic the structures and physical/chemical characteristics of the natural extracellular matrix and provide a suitable environment for cell proliferation and differentiation (Jahanbani et al., 2020). In the past decade, electrospinning has become an easy-to-operate and cost-effective method for fabricating tissue engineering scaffolds due to their unique ability to generate nanofibrous structures that mimics the natural morphology and function of the extracellular matrix (Zulkifli et al., 2023). Additionally, well-designed electrospun biomaterial scaffolds can have high surface area/volume ratios, and unique porosity to promote cell adhesion and proliferation (Liu Z. et al., 2020).

PCL is an FDA-approved synthetic polymer that has good biodegradability, biocompatibility and mechanical strength; however, its hydrophobicity often leads to poor cell adhesion, migration, growth and differentiation (Azari et al., 2022). Therefore, PCL could be combined with other types of biomaterials with hydrophilic and ionic characteristics to generate hybrid biomaterials and achieve improved tissue repair and regeneration outcomes. SF, which is a natural polymer extracted from silk, has been shown to be non-toxic, non-irritating, and it also has excellent flexibility, tensile resistance, air permeability, moisture permeability, biodegradability as well as good versatility of structural change (Sun et al., 2021). Different forms of SF, such as hydrogels, sponges, films, electrospun nanofiber pads and hydrocolloid dressings have been successfully used as wound dressings to promote the wound-healing process (Sultan et al., 2018). However, pure SF has often been modified or blended with other types of biomaterials in applications. Moreover, HA is an ECM-derived natural material that can regulate cell adhesion, proliferation and differentiation (Kotla et al., 2023). Additionally, HA alone has been previously used in IUA treatment as a mechanical and physical barrier to reduce intrauterine capillary bleeding and improve the intrauterine local tissue micro-environment (Ma et al., 2021). Despite its many advantages, HA has low viscosity, and HA might be used in combination with other types of biomaterials to promote cell adhesion, growth and tissue repair (Kim et al., 2019).

In this study, the effects of electrospun membranes made of PCL alone and PCL-based hybrid-biomaterials (PCL-SF, PCL-HA), on the adhesion, proliferation and inflammatory and wound-healing genes’ expression of H-EMSCs were investigated. The results showed that when compared with pure PCL electrospun membranes, PCL-SF and PCL-HA composite membranes had the advantages of both natural (SF or HA) and synthetic (PCL) materials and demonstrated higher biocompatibility and the ability to support the adhesion and proliferation of H-EMSCs. In addition, PCL-HA electrospun fibers supported higher adhesion and proliferation of H-EMSCs vs PCL-SF. It was further interesting to see that all three types of electrospun membranes (PCL, PCL-SF, PCL-HA) supported the mesenchymal stem cell genes CD90 and Meflin expression of the seeded H-EMSCs, suggesting that all the PCL-based electrospun membranes can maintain the mesenchymal stem cell phenotype of H-EMSCs during culture. However, the H-EMSCs cultured on PCL-HA electrospun membranes showed lower expression of proinflammatory gene IL-6 and higher expression of anti-inflammatory (IL-10) and wound-healing (VEGFA, TGF-β) genes vs PCL-SF, suggesting that HA could have the potential to better modulate the endometrial tissue inflammation and wound-healing vs SF. The reduction of IL-6 expression in the PCL-HA condition was of great importance as any approach that reduces chronic IL-6 release would be beneficial for cell proliferation and tissue development (Rose-John et al., 2023). Previous studies have shown that HA can modulate skin tissue inflammation and wound-healing processes by binding to fibrinogen to activate the clotting pathways, inhibiting neutrophil migration to decrease inflammation, and stimulating the secretion of matrix metalloproteinases for angiogenesis (Frenkel, 2014; Kaul et al., 2021; Kawano et al., 2021), which matched the proinflammation and wound-healing gene expression pattern of the H-EMSCs cultured on PCL-HA membrane observed in this study.

The reasons why PCL-HA is better than PCL-SF in cell adhesion experiments could include the following: Firstly, according to previous studies, HA has strong binding affinity to specific proteins, including CD44 (Li et al., 2023; Cheung et al., 2025) and intercellular adhesion molecule 1 (ICAM-1) (Shang et al., 2024), which are highly expressed on the surfaces of H-EMSCs (Wang et al., 2012; Darzi et al., 2016; Liu Y. et al., 2020; Abuwala and Tal, 2021). In addition, cell interaction with HA can lead to activation of the PI3K/Akt signaling pathway, which can promote actin remodeling to enhance cell adhesion and spreading (Mandal et al., 2019). Further, HA has been reported to exert anti-inflammatory functions in the endometrial tissue micro-environment (Zhou et al., 2019; Marinho et al., 2021), and the anti-inflammation environment (which could contain anti-inflammatory growth factors and cytokines) can promote the adhesion of H-EMSCs (Zhou et al., 2019). Interestingly, abundant HA has been detected in the uterine fluid and HA was demonstrated to play a critical role in enhancing the adhesion of embryos to the endometrial surface of the uterus (Rashki Ghaleno et al., 2024). Also, it should be realized that cell adhesion is a complex process, which could potentially be affected by many factors. The specific fiber structures and surface topography achieved in the electrospun PCL-HA membranes could have also played a role in enhancing H-EMSCs’ adhesion in comparison with PCL-SF membranes. Finally, it should be noted that different cell types could have various adhesion behaviors on PCL-HA and PCL-SF electrospun membranes, H-EMSCs (due to their expression of specific surface proteins such as CD 44 and ICAM-1) could prefer to adhere on PCL-HA membranes, while other cell types could be more likely to adhere on PCL-SF membranes. Therefore, the specific characteristics of the target cell type need to be considered when selecting biomaterials.

Although this study did not explore how HA affects the specific signaling pathways related to IL-6 or IL-10 expression, previous studies could provide some value and add to the strength of this study. For instance, it was found that HA can activate the PI3K/Akt/mTOR pathway, leading to enhanced IL-10 gene and protein expression in monocytes (Lenart et al., 2017). Additionally, HA also reduced the expression level of IL-6 in macrophages and chondrocytes by inhibiting the MAPK and p65/NF-κB signaling pathways (Kuppa et al., 2024). Further, researchers have found that HA inhibited the expression of TNF-α, IL-6, IL-1, and IFN-β, and enhanced the expression of IL-10 in human macrophages and inflammatory mice models (You et al., 2021). HA achieved those anti-inflammatory effects via inhibiting the phosphorylation of TLR4 signaling pathway proteins p65, IKKα/β, IKKBα, JNK1/2, ERK1/2, p38 and IRF-3 (You et al., 2021). Future studies in our group would deeply explore all of those potential signaling pathways that could modulate HA-induced IL-6 and IL-10 expression changes.

In recent years, although there have been some studies describing the use of H-EMSCs for IUA treatment, the therapeutic effect has been limited to date due in part to the low stem cell survival rate. Biomaterials have significant effects on the survival, migration, differentiation of stem cells, thereby can potentially enhance stem cell therapeutic effects and promote tissue regeneration (Mitrousis et al., 2018). Overall, in this study, PCL-HA is superior to PCL-SF for the following reasons. Firstly, the surface properties of PCL-HA electrospun membranes are more conducive to the adhesion and proliferation of H-EMSCs vs PCL-SF electrospun membranes. HA is a natural polysaccharide with good hydrophilicity and biocompatibility, and its molecular structure contains a large number of hydroxyl groups, which can bind to the receptors on the H-EMSC surface and promote cell adhesion and spreading (Li et al., 2023; Cheung et al., 2025). Additionally, PCL-HA downregulated IL-6 gene expression in H-EMSCs vs PCL-SF. In damaged endometrial tissue, overexpression of IL-6 may exacerbate the inflammatory response and affect tissue repair and regeneration (Mahmoud et al., 2024). The reduction of IL-6 gene expression by PCL-HA thereby could help to reduce inflammatory response and create a microenvironment that is more conducive to endometrial tissue healing (Incognito et al., 2023). Finally, PCL-HA upregulated the expression of IL-10, TGF-β and VEGFA genes in H-EMSCs vs PCL-SF. IL-10 is an anti-inflammatory cytokine that promotes tissue repair and regeneration (Ouyang and O’Garra, 2019). TGF-β can enhance cell proliferation, differentiation and migration, and also has a positive impact on tissue repair and regeneration (Zhang et al., 2017). VEGFA is a vascular endothelial growth factor that plays an important role in the formation and repair of blood vessels (Pérez-Gutiérrez and Ferrara, 2023). During the endometrial tissue repair and regeneration, the formation of blood vessels are essential to provide adequate blood supply for the regenerated endometrium (Hu et al., 2023). Therefore, PCL-HA is superior to PCL-SF in this study for endometrial tissue repair due to its ability to promote H-EMSCs’ adhesion, enhance anti-inflammation and angiogenesis.

Based on the findings of this study, an endometrial tissue patch generated by seeding H-EMSCs onto an electrospun PCL-HA membrane has great potential towards effectively treating IUA and could be transplanted into IUA disease models in future studies to further evaluate its potential towards modulating endometrial tissue inflammation and wound-healing processes *in vivo*. For *in vivo* animal model studies, we plan to develop a rat IUA model, and the PCL-HA electrospun membranes would be cut into rectangles of 2.5 cm × 0.5 cm and sterilized. 50 μL cell suspension containing 1 × 10^6^ H-EMSCs would be dropped on the PCL-HA electrospun membrane and incubated at 37°C in CO_2_ incubator for 24 h before use. The cell-seeded PCL-HA membranes would be implanted into the rat IUA model with the aid of a sterilized needle, the rat abdominal cavity would be closed layer by layer after the implantation surgery. For future clinical studies, the PCL-HA-H-EMSC patch probably needs to be delivered to the damaged endometrium with the use of a ballon catheter during hysteroscopic surgery.

At present, this study has started to develop IUA rat models for *in vivo* experiments and preliminary results have shown that PCL-HA membrane has anti-fibrosis effects in SD rat IUA models. However, the scaling up and clinical translation of the PCL-HA-H-EMSC patches face significant challenges. Firstly, the electrospinning parameters such as monomer/polymer concentration, electrospinning speed and electrical field strength need to be precisely regulated when fabricating the PCL-HA electrospun membranes, to ensure the uniformity and structural stability of the resulting electrospun membranes. At the same time, the isolation, culture and expansion of H-EMSCs needs to consider the purity, metabolic activity and functionality of the cells. Good manufacturing practices need to be followed for quality control and sterility during both the cell and material production processes. Clinical translation of the PCL-HA-H-EMSC patches needs to consider the regulatory issues and the marketing approval pathways are determined by the quality, safety and efficacy of the PCL-HA-H-EMSC patches being evaluated in both pre-clinical and clinical studies (such as toxicity, degradability and immunogenicity tests). In addition, it is critical to establish common international guidelines/regulations for the clinical application of PCL-HA-H-EMSC patches. Currently, for U.S., European union and China, medical devices are subjected to regulations from FDA, European Medicines Agency and China FDA respectively, which focus on different aspects of the products (quality, efficacy and utility). As a result, common international guidelines are essential to accelerate the clinical translation of the PCL-HA-H-EMSC patches. Further, it should be noted that the survival rate and retention time of H-EMSCs that are loaded on PCL-HA membranes can directly affect the IUA treatment effects in patients. Although PCL-HA electrospun membranes demonstrated ability to improve H-EMSCs’ survival and metabolic activity, this biomaterial-based electrospun membrane could be further optimized to ensure longer-term cell survival within patients’ endometrial tissue layer during the repairing process, enabling better therapeutic effects.

## 5 Conclusion

This study showed that PCL-HA electrospun membrane supported higher adhesion, proliferation and anti-inflammatory/wound-healing behaviors of H-EMSCs vs PCL and PCL-SF, suggesting the strong potential of a PCL-HA-H-EMSC patch being used to support the effective treatment of IUA. This study provided significant insights into the effects of synthetic/natural biomaterial combination on H-EMSCs proliferation and its potential ability to modulate inflammation and wound-healing.

## 6 Impact statement

Human endometrial MSCs showed enhanced adhesion and proliferation on PCL-HA vs PCL, PCL-SF, establishing the potential of the composite to address endometrial MSCs’ survival. H-EMSCs cultured on PCL-HA showed decreased IL-6 gene expression vs PCL-SF, eliminating a potential senescent pathway to improve cell anti-inflammatory property. H-EMSCs cultured on PCL-HA showed increased IL-10, VEGFA, TGF-β gene expression vs PCL-SF, establishing the potential to use H-EMSCs to create a favorable micro-environment for generating vascularized endometrial tissues. PCL, PCL-SF, PCL-HA all supported CD90 and Meflin expression of the seeded H-EMSCs, establishing PCL as a platform to form enhanced biomaterial composites for endometrial repair in the future.

## Data Availability

The original contributions presented in the study are included in the article/Supplementary Material, further inquiries can be directed to the corresponding authors.

## References

[B1] Abuwala N. Tal R. (2021). Endometrial stem cells: origin, biological function, and therapeutic applications for reproductive disorders. Curr. Opin. Obstet. Gynecol. 33, 232–240. 10.1097/GCO.0000000000000702 33896919 PMC9313610

[B2] Ang C. J. Skokan T. D. McKinley K. L. (2023). Mechanisms of regeneration and fibrosis in the endometrium. Annu. Rev. Cell Dev. Biol. 39, 197–221. 10.1146/annurev-cellbio-011723-021442 37843929 PMC11444732

[B3] Arabpour M. Saghazadeh A. Rezaei N. (2021). Anti-inflammatory and M2 macrophage polarization-promoting effect of mesenchymal stem cell-derived exosomes. Int. Immunopharmacol. 97, 107823. 10.1016/j.intimp.2021.107823 34102486

[B4] Ayran M. Dirican A. Y. Saatcioglu E. Ulag S. Sahin A. Aksu B. (2022). 3D-Printed PCL scaffolds combined with juglone for skin tissue engineering. Bioengineering 9, 427. 10.3390/bioengineering9090427 36134974 PMC9495790

[B5] Azari A. Golchin A. Maymand M. M. Mansouri F. Ardeshirylajimi A. (2022). Electrospun polycaprolactone nanofibers: current research and applications in biomedical application. Adv. Pharm. Bull. 12, 658–672. 10.34172/apb.2022.070 36415646 PMC9675923

[B6] Benor A. Gay S. DeCherney A. (2020). An update on stem cell therapy for Asherman syndrome. J. Assist. Reprod. Genet. 37, 1511–1529. 10.1007/s10815-020-01801-x 32445154 PMC7376809

[B7] Bozorgmehr M. Gurung S. Darzi S. Nikoo S. Kazemnejad S. Zarnani A. H. (2020). Endometrial and menstrual blood mesenchymal stem/stromal cells: biological properties and clinical application. Front. Cell Dev. Biol. 8, 497. 10.3389/fcell.2020.00497 32742977 PMC7364758

[B8] Cai H. Wu B. Li Y. Liu Y. Shi L. Gong L. (2019). Local delivery of silk-cellulose incorporated with stromal cell-derived factor-1α functionally improves the uterus repair. Tissue Eng. Part A 25, 1514–1526. 10.1089/ten.tea.2018.0283 30838933

[B9] Castro K. C. Campos M. G. N. Mei L. H. I. (2021). Hyaluronic acid electrospinning: challenges, applications in wound dressings and new perspectives. Int. J. Biol. Macromol. 173, 251–266. 10.1016/j.ijbiomac.2021.01.100 33476622

[B10] Cen J. Zhang Y. Bai Y. Ma S. Zhang C. Jin L. (2022). Research progress of stem cell therapy for endometrial injury. Mater Today Bio 16, 100389. 10.1016/j.mtbio.2022.100389 PMC9403503 36033375

[B11] Chen K. Li Y. Li Y. Pan W. Tan G. (2023). Silk fibroin combined with electrospinning as a promising strategy for tissue regeneration. Macromol. Biosci. 23, e2200380. 10.1002/mabi.202200380 36409150

[B12] Cheung B. C. H. Chen X. Davis H. J. Nordmann C. S. Toth J. Hodgson L. (2025). Identification of CD44 as a key engager to hyaluronic acid-rich extracellular matrices for cell traction force generation and tumor invasion in 3D. Matrix Biol. 135, 1–11. 10.1016/j.matbio.2024.11.004 39528207 PMC11729355

[B13] Darzi S. Werkmeister J. A. Deane J. A. Gargett C. E. (2016). Identification and characterization of human endometrial mesenchymal stem/stromal cells and their potential for cellular therapy. Stem Cells Transl. Med. 5, 1127–1132. 10.5966/sctm.2015-0190 27245365 PMC4996433

[B14] Debari M. K. King C. I. Altgold T. A. Abbott R. D. (2021). Silk fibroin as a green material. ACS Biomater. Sci. Eng. 7, 3530–3544. 10.1021/acsbiomaterials.1c00493 34260194

[B15] Emingr M. Halaj M. Malčák M. Hanáček J. (2023). Prevention of intrauterine adhesions. Ceska Gynekol. 88, 210–213. 10.48095/cccg2023210 37344187

[B16] Frenkel J. S. (2014). The role of hyaluronan in wound healing. Int. Wound J. 11, 159–163. 10.1111/j.1742-481X.2012.01057.x 22891615 PMC7950635

[B17] Fujii S. Miura Y. (2022). Immunomodulatory and regenerative effects of MSC-derived extracellular vesicles to treat acute GVHD. Stem Cells 40, 977–990. 10.1093/stmcls/sxac057 35930478

[B18] Grinchuk T. M. Shorokhova M. A. Pugovkina N. A. (2023). The response of the cell genome of endometrial mesenchymal stem cells to the procedure of long-term cyropreservation. Cell tissue Biol. 17, 627–638. 10.1134/S1990519X2306007X

[B19] Homaeigohar S. Boccaccini A. R. (2022). Nature-derived and synthetic additives to poly(ɛ-Caprolactone) nanofibrous systems for biomedicine; an updated overview. Front. Chem. 9, 809676. 10.3389/fchem.2021.809676 35127651 PMC8807494

[B20] Hong J. Yeo M. Yang G. H. Kim G. (2019). Cell-electrospinning and its application for tissue engineering. Int. J. Mol. Sci. 20, 6208. 10.3390/ijms20246208 31835356 PMC6940787

[B21] Hu X. Wu H. Yong X. Wang Y. Yang S. Fan D. (2023). Cyclical endometrial repair and regeneration: molecular mechanisms, diseases, and therapeutic interventions. MedComm (Beijing) 4, e425. 10.1002/mco2.425 PMC10691302 38045828

[B22] Incognito G. G. Di Guardo F. Gulino F. A. Genovese F. Benvenuto D. Lello C. (2023). Interleukin-6 as A Useful predictor of endometriosis-associated infertility: a systematic review. Int. J. Fertil. Steril. 17, 226–230. 10.22074/IJFS.2023.557683.1329 37577903 PMC10439985

[B23] Jahanbani Y. Davaran S. Ghahremani-Nasab M. Aghebati-Maleki L. Yousefi M. (2020). Scaffold-based tissue engineering approaches in treating infertility. Life Sci. 240, 117066. 10.1016/j.lfs.2019.117066 31738881

[B24] Kaul A. Short W. D. Keswani S. G. Wang X. (2021). Immunologic roles of hyaluronan in dermal wound healing. Biomolecules 11, 1234. 10.3390/biom11081234 34439900 PMC8394879

[B25] Kawano Y. Patrulea V. Sublet E. Borchard G. Iyoda T. Kageyama R. (2021). Wound healing promotion by hyaluronic acid: effect of molecular weight on gene expression and *in vivo* wound closure. Pharmaceuticals 14, 301. 10.3390/ph14040301 33800588 PMC8065935

[B26] Kim Y. Y. Park K. H. Kim Y. J. Kim M. S. Liu H. C. Rosenwaks Z. (2019). Synergistic regenerative effects of functionalized endometrial stromal cells with hyaluronic acid hydrogel in a murine model of uterine damage. Acta Biomater. 89, 139–151. 10.1016/j.actbio.2019.03.032 30898731

[B27] Kotla N. G. Mohd Isa I. L. Larrañaga A. Maddiboyina B. Swamy S. K. Sivaraman G. (2023). Hyaluronic acid-based bioconjugate systems, scaffolds, and their therapeutic potential. Adv. Healthc. Mater 12, e2203104. 10.1002/adhm.202203104 36972409

[B28] Kou L. Jiang X. Xiao S. Zhao Y. Z. Yao Q. Chen R. (2020). Therapeutic options and drug delivery strategies for the prevention of intrauterine adhesions. J. Control. Release 318, 25–37. 10.1016/j.jconrel.2019.12.007 31830539

[B29] Kundu B. Rajkhowa R. Kundu S. C. Wang X. (2013). Silk fibroin biomaterials for tissue regenerations. Adv. Drug Deliv. Rev. 65, 457–470. 10.1016/j.addr.2012.09.043 23137786

[B30] Kuppa S. S. Kang J. Y. Yang H. Y. Lee S. C. Sankaranarayanan J. Kim H. K. (2024). Hyaluronic acid viscosupplement modulates inflammatory mediators in chondrocyte and macrophage coculture via MAPK and NF-κB signaling pathways. ACS Omega 9, 21467–21483. 10.1021/acsomega.4c01911 38764654 PMC11097370

[B31] Lenart M. Rutkowska-Zapala M. Baj-Krzyworzeka M. Szatanek R. Węglarczyk K. Smallie T. (2017). Hyaluronan carried by tumor-derived microvesicles induces IL-10 production in classical (CD14++CD16−) monocytes via PI3K/Akt/mTOR-dependent signalling pathway. Immunobiology 222, 1–10. 10.1016/j.imbio.2015.06.019 26210045

[B32] Li L. Ding Q. Wu Y. Zheng Z. Zhang X. Zhang M. (2023). Binding of different hyaluronan to CD44 mediates distinct cell adhesion dynamics under shear flow. FEBS J. 290, 4695–4711. 10.1111/febs.16882 37254632

[B33] Li X. Lv H. F. Zhao R. Ying M. F. Samuriwo A. T. Zhao Y. Z. (2021). Recent developments in bio-scaffold materials as delivery strategies for therapeutics for endometrium regeneration. Mater Today Bio 11, 100101. 10.1016/j.mtbio.2021.100101 PMC8138682 34036261

[B34] Lin Y. Dong S. Ye X. Liu J. Li J. Zhang Y. (2022). Synergistic regenerative therapy of thin endometrium by human placenta-derived mesenchymal stem cells encapsulated within hyaluronic acid hydrogels. Stem Cell Res. Ther. 13, 66. 10.1186/s13287-022-02717-2 35135594 PMC8822809

[B35] Liu T. He B. Xu X. (2023). Repairing and regenerating injured endometrium methods. Reprod. Sci. 30, 1724–1736. 10.1007/s43032-022-01108-5 36653588

[B36] Liu Y. Liang S. Yang F. Sun Y. Niu L. Ren Y. (2020a). Biological characteristics of endometriotic mesenchymal stem cells isolated from ectopic lesions of patients with endometriosis. Stem Cell Res. Ther. 11, 346. 10.1186/s13287-020-01856-8 32771033 PMC7414689

[B37] Liu Z. Ramakrishna S. Liu X. (2020b). Electrospinning and emerging healthcare and medicine possibilities. Apl. Bioeng. 4, 030901. 10.1063/5.0012309 32695956 PMC7365682

[B38] Ma J. Zhan H. Li W. Zhang L. Yun F. Wu R. (2021). Recent trends in therapeutic strategies for repairing endometrial tissue in intrauterine adhesion. Biomater. Res. 25, 40. 10.1186/s40824-021-00242-6 34819167 PMC8611984

[B39] Mahmoud N. N. Hamad K. Al Shibitini A. Juma S. Sharifi S. Gould L. (2024). Investigating inflammatory markers in wound healing: understanding implications and identifying artifacts. ACS Pharmacol. Transl. Sci. 7, 18–27. 10.1021/acsptsci.3c00336 38230290 PMC10789122

[B40] Mandal K. Raz-Ben Aroush D. Graber Z. T. Wu B. Park C. Y. Fredberg J. J. (2019). Soft hyaluronic gels promote cell spreading, stress fibers, focal adhesion, and membrane tension by phosphoinositide signaling, not traction force. ACS Nano 13, 203–214. 10.1021/acsnano.8b05286 30500159 PMC6511072

[B41] Manoukian O. S. Matta R. Letendre J. Collins P. Mazzocca A. D. Kumbar S. G. (2017). “Electrospun nanofiber scaffolds and their hydrogel composites for the engineering and regeneration of soft tissues,” in Methods in molecular biology. 10.1007/978-1-4939-6840-4_18 28238143

[B42] Marinho A. Nunes C. Reis S. (2021). Hyaluronic acid: a key ingredient in the therapy of inflammation. Biomolecules 11, 1518. 10.3390/biom11101518 34680150 PMC8533685

[B43] Maurmann N. França F. S. Girón J. Pranke P. (2023). Cell electrospinning: a review of materials and methodologies for biofabrication. Adv. Biol. 7, e2300058. 10.1002/adbi.202300058 37271854

[B44] Mitrousis N. Fokina A. Shoichet M. S. (2018). Biomaterials for cell transplantation. Nat. Rev. Mater 3, 441–456. 10.1038/s41578-018-0057-0

[B45] Ng S. W. Norwitz G. A. Pavlicev M. Tilburgs T. Simón C. Norwitz E. R. (2020). Endometrial decidualization: the primary driver of pregnancy health. Int. J. Mol. Sci. 21, 4092. 10.3390/ijms21114092 32521725 PMC7312091

[B46] Ouyang W. O’Garra A. (2019). IL-10 family cytokines IL-10 and IL-22: from basic science to clinical translation. Immunity 50, 871–891. 10.1016/j.immuni.2019.03.020 30995504

[B47] Pérez-Gutiérrez L. Ferrara N. (2023). Biology and therapeutic targeting of vascular endothelial growth factor A. Nat. Rev. Mol. Cell Biol. 24, 816–834. 10.1038/s41580-023-00631-w 37491579

[B48] Rahimipour M. Jafarabadi M. Salehnia M. (2021). *In vitro* implantation model using human endometrial SUSD2+ mesenchymal stem cells and myometrial smooth muscle cells. Cell J. 23, 154–163. 10.22074/cellj.2021.6979 34096216 PMC8181319

[B49] Rashki Ghaleno L. Pennisi C. P. Shahverdi A. Dardmeh F. Alipour H. Rezazadeh Valojerdi M. (2024). Exploring the role of hyaluronic acid in reproductive biology and beyond: applications in assisted reproduction and tissue engineering. Adv. Biol. 8, 2300621. 10.1002/adbi.202300621 38580620

[B50] Rose-John S. Jenkins B. J. Garbers C. Moll J. M. Scheller J. (2023). Targeting IL-6 trans-signalling: past, present and future prospects. Nat. Rev. Immunol. 23, 666–681. 10.1038/s41577-023-00856-y 37069261 PMC10108826

[B51] Shang L. Li M. Xu A. Zhuo F. (2024). Recent applications and molecular mechanisms of hyaluronic acid in skin aging and wound healing. Med. Nov. Technol. Devices 23, 100320. 10.1016/j.medntd.2024.100320

[B52] Sultan M. T. Lee O. J. Kim S. H. Ju H. W. Park C. H. (2018). “Silk fibroin in wound healing process,” in Advances in experimental medicine and biology. 10.1007/978-981-13-0947-2_7 30357686

[B53] Sun W. Gregory D. A. Tomeh M. A. Zhao X. (2021). Silk fibroin as a functional biomaterial for tissue engineering. Int. J. Mol. Sci. 22, 1499. 10.3390/ijms22031499 33540895 PMC7867316

[B54] Vasvani S. Kulkarni P. Rawtani D. (2020). Hyaluronic acid: a review on its biology, aspects of drug delivery, route of administrations and a special emphasis on its approved marketed products and recent clinical studies. Int. J. Biol. Macromol. 151, 1012–1029. 10.1016/j.ijbiomac.2019.11.066 31715233

[B55] Vitale S. G. Riemma G. Carugno J. Perez-Medina T. Alonso Pacheco L. Haimovich S. (2022). Postsurgical barrier strategies to avoid the recurrence of intrauterine adhesion formation after hysteroscopic adhesiolysis: a network meta-analysis of randomized controlled trials. Am. J. Obstet. Gynecol. 226, 487–498.e8. 10.1016/j.ajog.2021.09.015 34555319

[B56] Wang H. Jin P. Sabatino M. Ren J. Civini S. Bogin V. (2012). Comparison of endometrial regenerative cells and bone marrow stromal cells. J. Transl. Med. 10, 207. 10.1186/1479-5876-10-207 23038994 PMC3504519

[B57] Wang L. Yu C. Chang T. Zhang M. Song S. Xiong C. (2020a). *In situ* repair abilities of human umbilical cord-derived mesenchymal stem cells and autocrosslinked hyaluronic acid gel complex in rhesus monkeys with intrauterine adhesion. Sci. Adv. 6, eaba6357. 10.1126/sciadv.aba6357 32494750 PMC7244313

[B58] Wang Y. Nicholes K. Shih I. M. (2020b). The origin and pathogenesis of endometriosis. Annu. Rev. Pathology Mech. Dis. 15, 71–95. 10.1146/annurev-pathmechdis-012419-032654 PMC7980953 31479615

[B59] You N. Chu S. Cai B. Gao Y. Hui M. Zhu J. (2021). Bioactive hyaluronic acid fragments inhibit lipopolysaccharide-induced inflammatory responses via the Toll-like receptor 4 signaling pathway. Front. Med. 15, 292–301. 10.1007/s11684-020-0806-5 32946028

[B60] Zhang Y. Alexander P. B. Wang X. F. (2017). TGF-Β family signaling in the control of cell proliferation and survival. Cold Spring Harb. Perspect. Biol. 9, a022145. 10.1101/cshperspect.a022145 27920038 PMC5378054

[B61] Zhang Z. Gupte M. J. Ma P. X. (2013). Biomaterials and stem cells for tissue engineering. Expert Opin. Biol. Ther. 13, 527–540. 10.1517/14712598.2013.756468 23327471 PMC3596493

[B62] Zheng Y. Li L. Bi X. Xue R. (2022). circPTP4A2-miR-330-5p-PDK2 signaling facilitates *in vivo* survival of HuMSCs on SF-SIS scaffolds and improves the repair of damaged endometrium. Oxid. Med. Cell Longev. 2022, 1–14. 10.1155/2022/2818433 PMC9106474 35571241

[B63] Zhou W. J. Yang H. L. Shao J. Mei J. Chang K. K. Zhu R. (2019). Anti-inflammatory cytokines in endometriosis. Cell. Mol. Life Sci. 76, 2111–2132. 10.1007/s00018-019-03056-x 30826860 PMC11105498

[B64] Zulkifli M. Z. A. Nordin D. Shaari N. Kamarudin S. K. (2023). Overview of electrospinning for tissue engineering applications. Polym. (Basel) 15, 2418. 10.3390/polym15112418 PMC10255387 37299217

[B65] Zuo W. Xie B. Li C. Yan Y. Zhang Y. Liu W. (2018). The clinical applications of endometrial mesenchymal stem cells. Biopreserv Biobank 16, 158–164. 10.1089/bio.2017.0057 29265881 PMC5906727

