# Supplementary figures and images for: Defining optimal electrospun membranes to enhance biological activities of human endometrial MSCs
Source: Front Bioeng Biotechnol. 2025 Feb 26;13:1551791. doi: 10.3389/fbioe.2025.1551791 (PMC11896994; doi:10.3389/fbioe.2025.1551791)

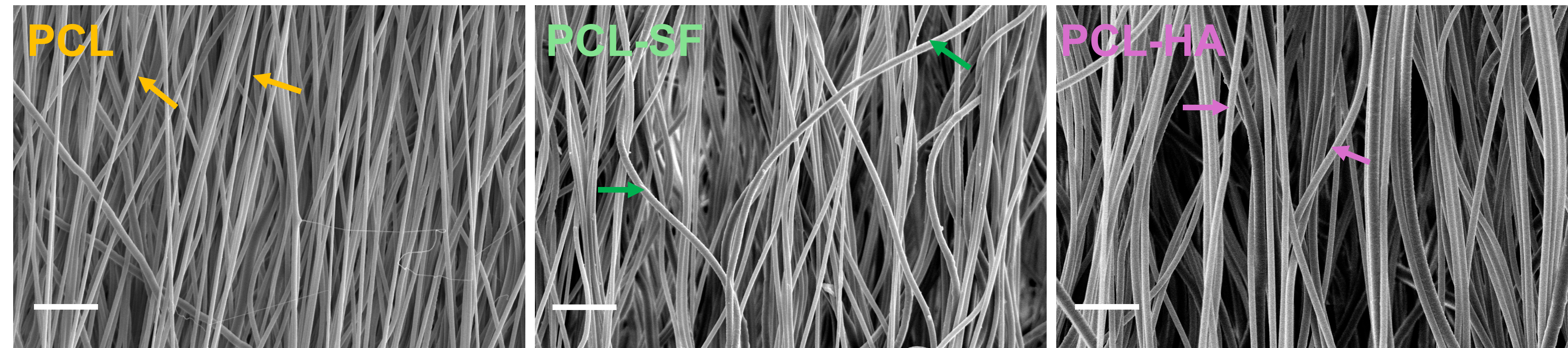

Supplement: Supplementary file 1 [file Image1.tif]
